# Anticancer Activity of Fucoidan via Apoptosis and Cell Cycle Arrest on Cholangiocarcinoma Cell

**DOI:** 10.31557/APJCP.2021.22.1.209

**Published:** 2021-01

**Authors:** Pathanin Chantree, Kesara Na-Bangchang, Pongsakorn Martviset

**Affiliations:** 1 *Division of Anatomy, Department of Preclinical Science, Faculty of Medicine, Thammasat University, Pathumthani, 12120, Thailand. *; 2 *Research Unit in Nutraceuticals and Food Safety, Faculty of Medicine, Thammasat University, Pathumthani, 12120, Thailand. *; 3 *Graduate Program in Bioclinical Sciences, Chulabhorn International College of Medicine, Thammasat University, Pathumthani, 12120, Thailand. *; 4 *Center of Excellence in Molecular Biology and Pharmacology of Malaria and Cholangiocarcinoma, Thammasat University, Pathumthani, 12120, Thailand. *; 5 *Division of Parasitology, Department of Preclinical Science, Faculty of Medicine, Thammasat University, Pathumthani, 12120, Thailand. *

**Keywords:** Fucoidan, apoptosis, cell cycle, CL-6- OUMS, cholangiocarcinoma cell

## Abstract

**Objective:**

Many previous studies reported that fucoidan has antitumor activities. The objective of the present study was to determine the cytotoxic effects and related mechanisms of cell death induced by fucoidan extracted from* Fucus vesiculosus* on CL-6 cholangiocarcinoma cell.

**Methods:**

CL-6 and OUMS cells were treated with 0, 100, 200, and 300 μg/mL of fucoidan. MTT assay was used to determine cytotoxicity. Flow cytometry-based assay was used to examine the distribution of apoptosis and cell cycle. The changes in nuclear morphology were determined using Hoechst 33,342 staining. Mitochondrial membrane potential (ΔΨm) was evaluated using the JC-1 kit. The apoptotic, anti-apoptotic, and cell cycle-related proteins study were examined by Western blot analysis.

**Results:**

The relative viable cell number of treated CL-6 cells was decreased but no effect was observed in OUMS normal cells. Furthermore, treated cells were arrested in the G0/G1 phase with down-regulation of cyclin D1 and CDK4. Annexin V/PI staining with flow cytometry analysis suggested that fucoidan could induce apoptosis in CL-6 cells. Western blot study revealed the up-regulation of apoptotic markers including Bax, cleaved PARP, cleaved caspase-3, but down-regulation of anti-apoptotic markers, cl-2. Moreover, fucoidan could induce nuclear fragmentation and chromatin condensation with alteration of ΔΨm.

**Conclusion:**

Fucoidan exerts antitumor properties against CL-6 cholangiocarcinoma cells illustrated by the induction of apoptosis and cell cycle arrest.

## Introduction

Cholangiocarcinoma (CCA) is a highly offensive malignancy arising from the epithelium of the biliary tract which consists of intrahepatic (iCCA) and extrahepatic cholangiocarcinoma (eCCA) (Valle et al., 2016). In general, the cell surface receptor tyrosine kinase c-Met only presents in progenitor and stem cells involved in organogenesis and wound healing. However, this receptor is anomalously high in cholangiocarcinoma along with its ligand hepatocyte growth factor (HGF) leading to enhance cell proliferation, angiogenesis, and metastasis (Socoteanu et al., 2008; Leelawat et al., 2006). Moreover, a key molecule in angiogenesis, vascular endothelial growth factor (VEGF), is highly expressed in cholangiocarcinoma both in the tissue samples and cell lines (Ogasawara et al., 2001). This molecular characteristic is facilitated by the presentation of the estrogen receptors that respond to 17-β estradiol resulting in increased VEGF production and angiogenesis (Alvaro et al., 2006; Mancino et al., 2009). 

In most countries, CCA is considered as low incidence cancer, on the contrary, a higher incidence has been reported from Southeast Asian countries especially in Laos PDR, Cambodia, Vietnam, and also Northeastern Thailand related to liver fluke, *Opisthorchis viverrini*, infection which has an incidence of more than 110/100,000 population (Bergquist and von Seth, 2015). Because of the late manifestation of symptoms and lack of early diagnostic tools, most patients present with metastasis results in less than 10% survival rate at 5 years (Bertuccio et al., 2019). The retrospective multicenter study demonstrated a 5-year survival of 73% after liver transplantation in patients with very early CCA defined as single tumors ≤ 2 cm in diameter (Sapisochin et al., 2014). However, a follow-up study with an international multicenter cohort of patients found a 5-year survival of 65% in patients with very early CCA (Sapisochin et al., 2016). 

Drug development is another backbone of CCA treatment due to ineffective chemotherapy. The current standard chemotherapy for CCA is including gemcitabine or 5-fluorouracil (5-FU) – based regimens, unluckily, it has high resistance. There are more than 100 genes involved in chemoresistance that have been identified and classified based on their mechanism of action on CCA including impaired drug uptake, enhanced mechanisms of DNA repair, and detoxifying capability. Moreover, CCA has the ability in enhanced resistance to apoptosis, change in the tumor environment and its phenotype (Fouassier et al., 2019; Khan et al., 2012; Park et al., 2015; Ramírez-Merino et al., 2013; Valle et al., 2010). Consequently, the development of effective chemotherapeutic agents with fewer side effects especially alimentary tract mucositis and immunosuppression (Bertolini et al., 2017; Lee et al., 2016) is still demanded. 

Fucoidan is a natural sulfated polysaccharide extracted from brown seaweed (Rodríguez-Jasso et al., 2013) and sea cucumber (Mansour et al., 2019). It mainly comprises fucose with below 10% of monosaccharides (Li et al., 2008; Mabeau et al., 1990). Fucoidan that is extracted from *Fucus vesiculosus* contains a backbone of α-(1 → 3)-linked fucose and α-(1 → 4)-linked fucose residues. Sulfation occurs mainly at *O*-2 and to a lesser extent at *O*-3. Also, 2, 3-*O*-disulfate fucose residues were sometimes found. The sulfate groups and the 2, 3-*O*-disulfate fucose of fucoidan molecules are important for various bioactivities especially the anti-cancer activity (Geert et al., 2019). Moreover, previous studies have shown that fucoidan exerts anti-inflammatory (Phull and Kim, 2017), antioxidant (Hou et al., 2012; Imbs et al., 2014), anti-viral (Dinesh et al., 2016), anti-bacterial (Back et al., 2010), anticoagulant effects (Mansour et al., 2019), and anti-diabetes effects (Jeong et al., 2013). 

As mentioned previously, the unique characteristic of cholangiocarcinoma is uncontrolled division, apoptotic resistance, aggressive metastasis, and massive neovascularization (Socoteanu et al., 2008; Leelawat et al., 2006). Hence, developing chemotherapeutic agents with potent anticancer capability should have an inhibitory efficacy to those characteristics. Fucoidan has enhanced anticancer effects via apoptotic induction both *in vitro* and *in vivo*. Previous *in vitro* studies reported that fucoidan induced chromatin condensation and cleavage of poly (ADP-ribose) polymerase (PARP) in the colon cancer cell (Kim et al., 2010), down-regulated anti-apoptotic proteins, and induced autophagy in gastric cancer cells (Park et al., 2011), activated caspase and cell cycle arrest in breast cancer cells (Banafa et al., 2013), disturbed mitochondrial membrane potential in melanoma (Chen et al., 2008), inhibited the production of VEGF, by which suppressing the angiogenesis in lung carcinoma cells (Huang et al., 2015). Previous *in vivo* studies suggested that fucoidan suppressed the metastasis of Lewis lung adenocarcinoma (Alekseyenko et al., 2007), the angiogenesis of prostate (Rui et al., 2017) and breast cancers (Xue et al., 2012), the growth of rat mammary adenocarcinoma (Coombe et al., 1987) and Ehrlich ascites carcinoma (Zhuang et al., 1995). Moreover, a previous study in mice that were transplanted with acute promyelocytic leukemia cells, fucoidan enhanced the killing activity of NK cells and suppressed the growth of cancer (Atashrazm et al., 2016). Nevertheless, there is no evaluation of the anticancer effect of fucoidan in CAA.

In this study, we determined the anticancer effects with related molecules of fucoidan extracted from *Fucus vesiculosus* in CL-6 cholangiocarcinoma cells. The results furnish beneficial evidence for the application of fucoidan for further cholangiocarcinoma treatment. 

## Materials and Methods

*Fucoidan and cell culture*

Fucoidan from *Fucus vesiculosus* was purchased from Sigma–Aldrich, USA. The stock solution of fucoidan was prepared by dissolving in phosphate-buffered saline (PBS) and stored at −20°C. The CCA cell line (CL-6) was kindly provided by Associate Professor Dr. Adisak Wongkajornsilp, Faculty of Medicine, Siriraj Hospital, Mahidol University, Thailand. Human embryonic fibroblast cell line (OUMS) was purchased from Japanese Collection of Research Bioresources (JCRB) cell bank, Japan. CL-6 cells were cultured in RPMI medium and OUMS cells were culture in DMEM medium. Both culture media were supplemented with 1% penicillin-streptomycin (10,000 U/mL) and 10% heat-inactivated fetal bovine serum (Gibco™, Thermo Fisher Scientific, Gaithersburg, MD, USA), and cell cultures were maintained under 5% CO_2_ atmosphere at 37°C.

*Cytotoxicity analysis*

CL-6 cells were seeded into 96-well plates and incubated under 5% CO_2_ atmosphere at 37°C for 24 h. Next, cells were incubated with fucoidan diluted in PBS in 0, 50, 100, 150, 200, 250, 300, 350, and 400 µg/mL for 24 h. Thereafter, the 30 μg/mL MTT working solution (Sigma-Aldrich, Saint Louis, MO, USA) was added into each well and further incubated for 3 h. The optical density (O.D.) values were measured by using a spectrophotometer at 562 and 630 nm and the results were used to evaluate the half-maximal inhibitory concentration (IC_50_) value. Thereafter, CL-6 and OUMS cells were incubated with fucoidan including the concentration of approximately 0.5X IC_50_ (100 μg/mL), IC_50_ (200 μg/mL), and 1.5X IC_50_ (300 μg/mL) for 24, 48, and 72 h. The cytotoxicity effect was determined by the MTT assay. These three concentrations of fucoidan were used for further methods. The experiments were done three-independence with triplicate each.

*Annexin V/PI apoptosis assay*

CL-6 and OUMS cells were incubated with fucoidan for 48 h under 5% CO_2_ atmosphere at 37°C. Thereafter, 1 × 10^5^ harvested cells were evaluated the apoptotic stages by using FITC Annexin V Apoptosis Detection Kit I (BD Pharmingen™, Franklin Lakes, NJ, USA). Briefly, cells were incubated with FITC Annexin V and PI in the binding buffer for 15 min at room temperature in the dark. The BD FACSCalibur™ flow cytometry system (BD Biosciences, San Jose, CA, USA) was used to examine the distribution of apoptotic stages of treated cells. 

*Nuclear Staining*

The chromosome condensation and fragmentation were evaluated by staining with Hoechst 33342 (Sigma-Aldrich, Saint Louis, MO, USA). After incubation with fucoidan, the culture media were removed and the cells were washed with PBS three times. After that, treated cells were incubated with 1:2,000 dilution of 10 mg/mL Hoechst 33342 stock solution and incubated for 15-20 min at room temperature in a dark condition. Lastly, cells were washed three times in PBS and examined by a fluorescence microscope at 480 nm.

*Evaluation of mitochondrial membrane potential*

Mitochondrial membrane potential was observed by using the JC-1 mitochondria staining Kit (Sigma-Aldrich, Saint Louis, MO, USA). Treated CL-6 cells were incubated with a JC-1 staining solution for 20 min in the dark under 5% CO_2_ atmosphere at 37°C. Thereafter, cells were washed three times and overlaid with a culture medium. JC-1 red and green fluorescence intensities were measured with Varioskan™ LUX Multimode Microplate Reader (Thermo Fisher Scientific Inc., USA). The ratio of the JC-1 with red fluorescence (aggregate form) to JC-1 green fluorescence (monomer form) for each treatment was calculated. 

Furthermore, CL-6 cells (1 × 10^5^) were seeded in 6-well plates for 24 h and subsequently incubated with fucoidan for 48 h under 5% CO_2_ atmosphere at 37°C. Thereafter, cells were incubated with JC-1 staining solution for 20 min at 37°C, washed three times with a growth medium, and examined under the fluorescence microscope.

*Flow cytometry analysis of cell cycle distribution*

Treated cells were evaluated cell cycle distribution by using propidium iodide. After incubated with fucoidan under 5% CO_2_ atmosphere at 37°C for 48 h, 2 × 10^6^ treated cells were fixed with 70% ethanol and incubated in a -20°C for 24 h. Thereafter, the ethanol was removed by washing cells three times in PBS. Then, cells were re-suspended in PBS containing 40 mg/mL of propidium iodide with 100 mg/mL of DNase-free RNase A (Sigma-Aldrich, Saint Louis, MO, USA) and incubated for 15 min at room temperature in the dark. The DNA content was evaluated using the BD FACSCalibur™ flow cytometry system (BD Biosciences, San Jose, CA, USA).

*Western blot analysis*

After incubation with fucoidan, total cellular proteins were extracted by using RIPA cell lysis buffer (Cell Signaling Technolog^®^, USA) containing protease inhibitors (Merck Millipore Calbiochem™ Protease Inhibitor Cocktail Set III, EDTA-Free, Germany). The protein concentrations were measured by using Pierce™ BCA Protein Assay Kit (Thermo Fisher Scientific Inc., Rockford, IL, USA). 30 μg of total proteins from each sample were size-separated on 12.5% SDS-PAGE and subsequently transferred onto nitrocellulose membranes. The non-specific bindings were blocked by using 5% BSA in tris-buffered saline (TBS), pH 7.5 for 1 h at room temperature with agitation. The membranes were then incubated with 1:2,000 primary antibodies diluted in 1% BSA in TBS with Tween^®^-20 (TBST) including rabbit anti-β-actin, rabbit anti-Bcl-2, rabbit anti-Bax, rabbit anti-cleaved PARP, rabbit anti-cleaved caspase-3, rabbit anti-cyclin D1, and rabbit anti-CDK4 (Cell Signaling Technolog^®^, USA) at 4°C overnight with agitation. Thereafter, the membranes were washed three times in TBST and further incubated with 1:15,000 horseradish peroxidase (HRP) conjugated goat anti-rabbit secondary antibody (Abcam, USA) diluted in 0.01M TBST for 1 h at room temperature. The corresponding targeted proteins were visualized by using TMB substrate (Thermo Fisher Scientific Inc., USA). Protein bands’ intensity was quantified by using ImageJ software.

*Statistical analysis*

Data were expressed as the mean ± SD obtained from triplicate experiments. Statistical analysis was performed using one-way analysis of variance (ANOVA) in SPSS 22. The values obtained in the assays were considered to be statistically significant when P < 0.05.

## Results

*Cytotoxicity of fucoidan on CL-6 and OUMS cells*

**Figure 1:**
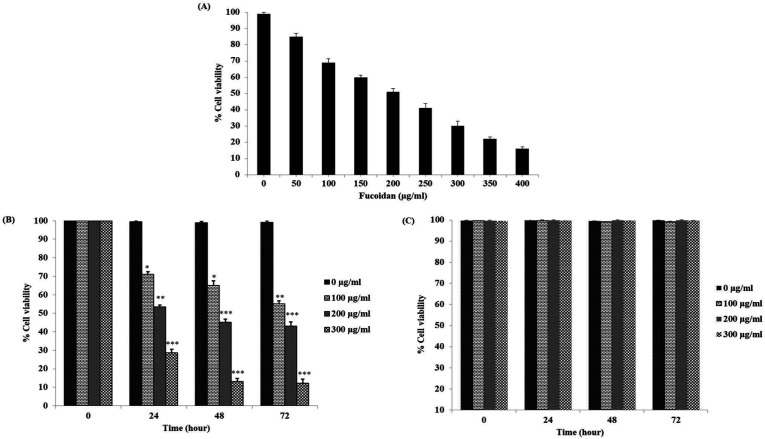
(A) Percent of cell viability of CL-6 cells after incubation with the fucoidan in the concentrations range from 50 to 400 µg/mL for 24 h. Thereafter, the IC50 value of fucoidan was evaluated. Cytotoxic effects of a fucoidan from 0 to 300 µg/mL on (B) CL-6 cells and (C) OUMS cells were examined at different time points. All values are presented in mean ± SD.*p<0.05, ** p < 0.01, ***p < 0.001

After treatment for 24 h with fucoidan, cell viability percents of CL-6 cells were decreased in a concentration-dependent manner from 50 to 400 μg/mL compared with the untreated group (Figure 1A). The 50% inhibitory concentration (IC_50_) of fucoidan for CL-6 cells was 196.27 ± 3.42 μg/mL. 

According to IC_50_, CL-6 and OUMS cells were subsequently treated with fucoidan (0, 100, 200, and 300 μg/mL) for 24, 48, and 72 h. Cell viability was examined using the MTT assay. Fucoidan treatment revealed decrease cell viability in CL-6 cells in a concentration-dependent manner (Figure 1B) but in OUMS cells revealed no effects (Figure 1C). The percentage of viability at various concentrations was calculated as the relative cell viability (%) of viable treated cells to viable control cells. 

*Analysis of apoptotic cells by flow cytometry in CL-6 and OUMS cells*

**Figure 2:**
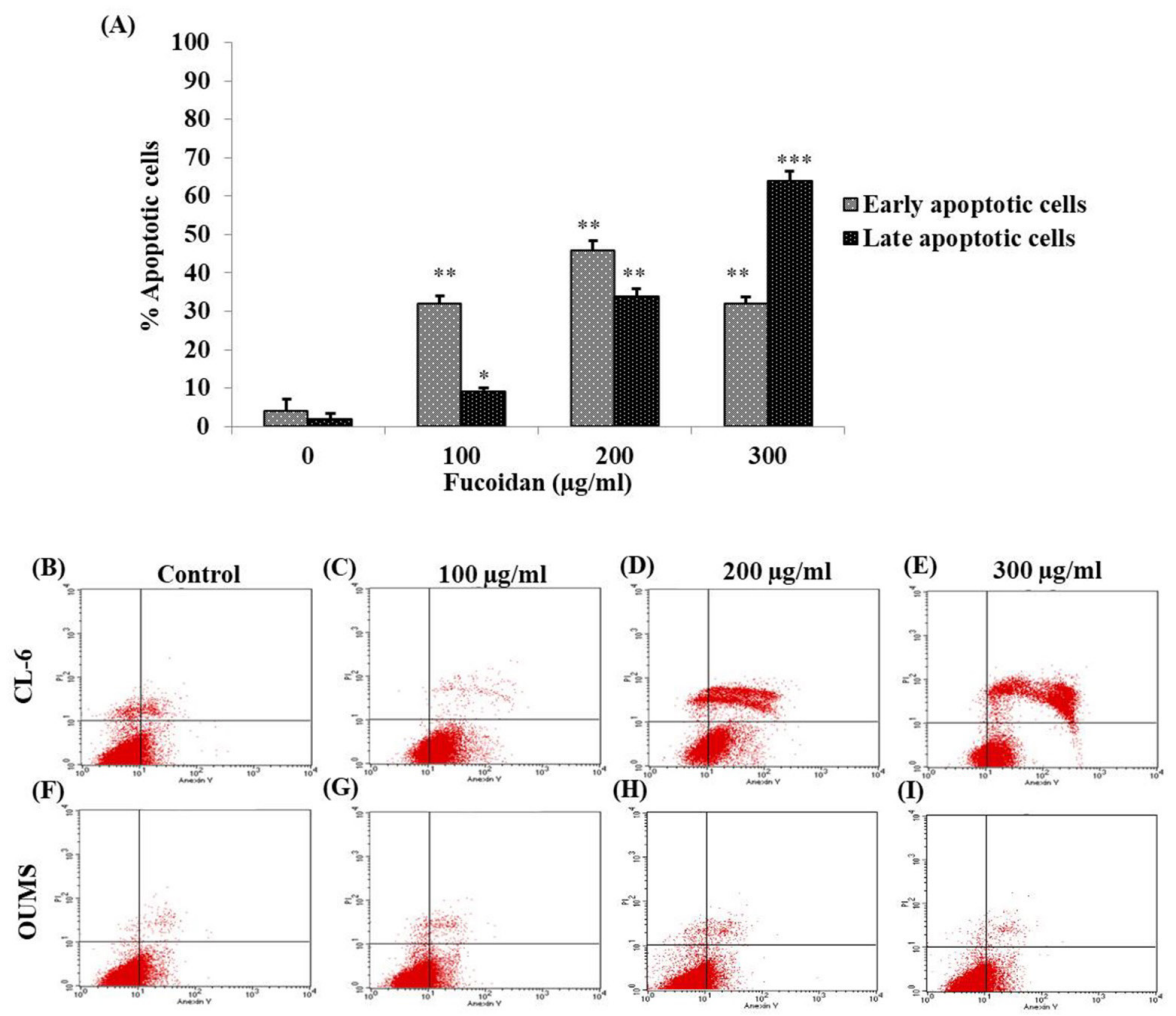
Apoptotic Induction of Fucoidan in CL-6 and OUMS Cells was Determined by Using Flow Cytometry. (A) The percentage of CL-6 cells are disseminated in early and late apoptotic stages per total cells. The numbers of apoptotic CL-6 cells (B-E) and OUMS cells (F-I) that were incubated in 0, 100, 200, and 300 μg/mL of fucoidan are illustrated in the scattered plot. *p < 0.05, ** p < 0.01, ***p < 0.001

The disruption of the cell membrane of apoptosis cells can cause Annexin V and propidium iodide infiltration to the cells. In the flow cytometry analysis, the results illustrated that numerous Annexin V positive cells can be observed in treated CL-6 cells that were higher than in the control group (Figure 2B, C, D, and E). Moreover, the percentage of treated cells distributed in early and late apoptotic phases were significantly increased in a concentration-dependent manner (Figure 2A). On the contrary, the number of Annexin V positive cells in treated OUMS cells was not different when compared to control (Figure 2F, G, H, and I). The present study confirmed that fucoidan could induce apoptosis in CL-6 cells. 

*Nuclear morphology observation of CL-6 cells*

**Figure 3:**
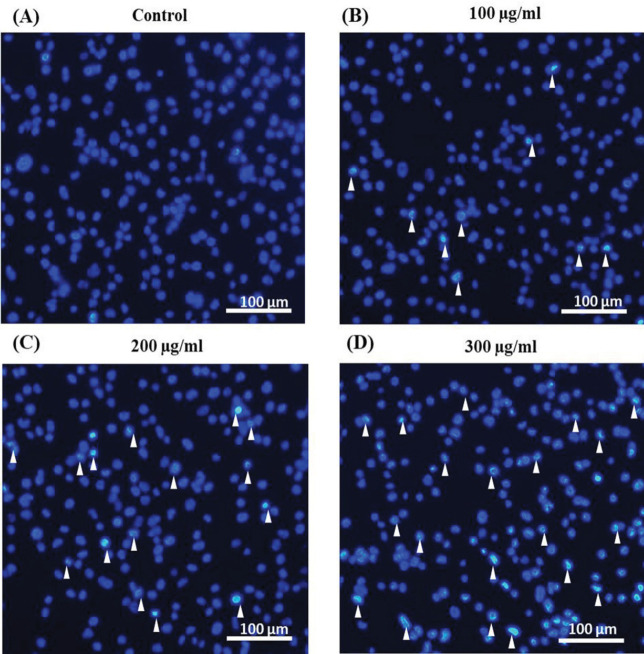
The Observation of Nuclear Morphology Changes in Treated CL-6 Cells (A-D). The results illustrated the nuclei stained with Hoechst 33342 after incubated with 0, 100, 200, and 300 μg/mL of fucoidan, respectively. Arrowheads represent nuclear fragmentation and chromatin condensation Scale bar = 100 μm

The nuclear morphological changes of treated cells were examined by Hoechst 33342 staining. After incubation with fucoidan, the bright bluish fluorescence nuclei with chromatin condensation and nuclear fragmentation which refer to nuclear damage were illustrated in treated cells (Figure 3B, C, and D) whereas uniform staining nuclei were observed in the control cells (Figure 3A). Employing the nuclear damage effect, it suggested that fucoidan could lead CL-6 cells to undergo apoptosis.

*Evaluation of mitochondrial membrane potential of CL-6 cells*

**Figure 4:**
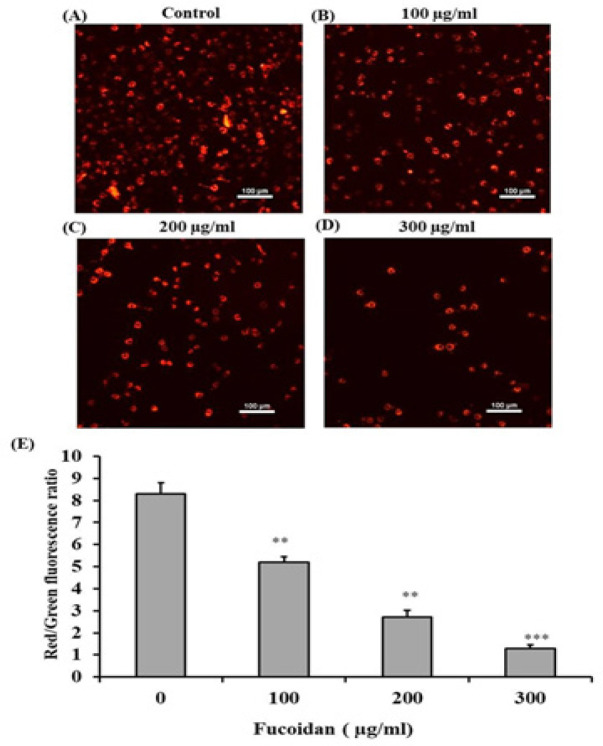
Disruption of the Mitochondrial Membrane Potential of Fucoidan in Treated CL-6 Cells. (A-D) The examination under the fluorescence microscope suggested the reduced number of CL-6 cells with red fluorescence in a dose-dependent manner from 0-300 μg/mL of fucoidan. (E) The red to green fluorescence ratio of JC-1 was significantly reduced in the dose-dependent manner (* p < 0.05, ** p < 0.01, ***p < 0.001). Scale bar = 100 μm

The effect of fucoidan on mitochondrial membrane potential (ΔΨm) of CL-6 cells was determined by using JC-1 staining. In normal cells, the JC-1 dye forms aggregation which emits red fluorescence in healthy mitochondria. In apoptotic cells, the JC-1 dye stays in a monomer which emits green fluorescence and implies low ΔΨm. Consequently, the mitochondrial depolarization is demonstrated by a decrease in the ratio of red to green fluorescence intensity. Treatment with the increasing concentrations of fucoidan resulted in the decreased number of CL-6 cells with red fluorescence in a concentration-dependent manner (Figure 4A, B, C, and D). The results also revealed a significant reduction in the JC-1 ratio (Figure 4E). These results suggested that fucoidan altered the mitochondrial membrane potential and induced apoptosis in CL-6 cells.

*Effect of fucoidan on the cell cycle of CL-6 cells*

**Figure 5:**
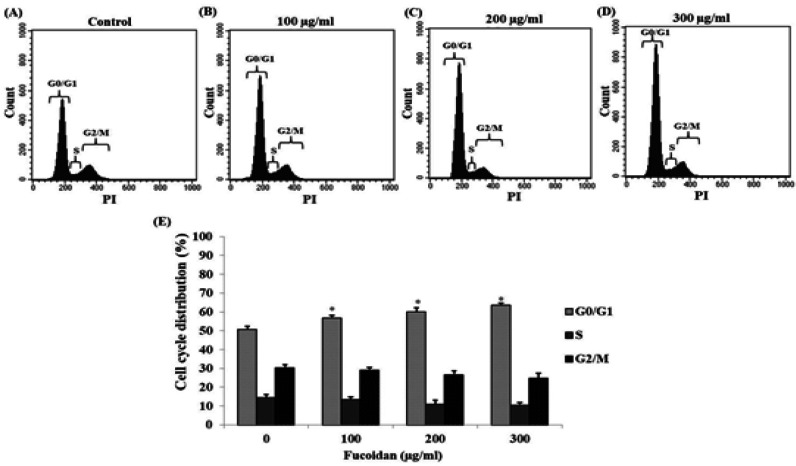
The Treated CL-6 Cells were Determined the Effect of Fucoidan on the Cell Cycle. The results revealed the distribution of CL-6 cells in various phases of the cell cycle (A): control group, (B) 100 μg/mL, (C) 200 μg/mL, and (D) 300 μg/mL fucoidan-treated groups, respectively. (E) Percentage of cells in different phases was presented as mean ± SD.* p < 0.05

After CL-6 cells were incubated with various concentrations of fucoidan, cells were stained with propidium iodide and analyzed the phase distribution in the cell cycle using flow cytometry. The results suggested that fucoidan could induce an arrest of CL-6 cells in the G0/G1 phase (Figure 5A, B, C, and D). The number of arrested cells was significantly increased in a concentration-dependent manner in the G0/G1 phase. However, the ratios of cells in S and G2/M phase were not significantly decreased (Figure 5E).

**Figure 6:**
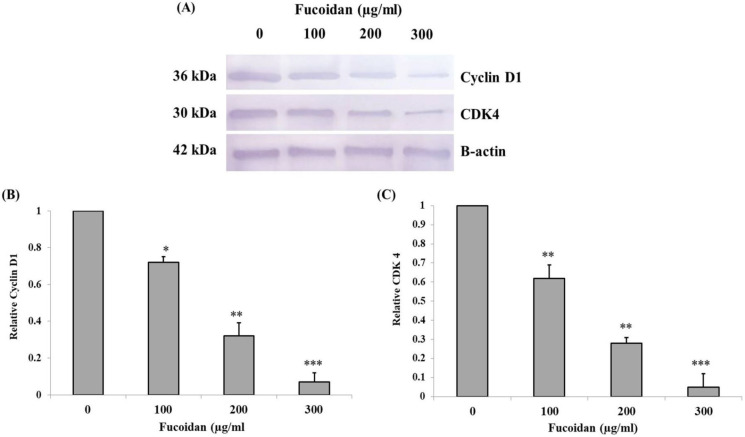
Western Bblot Analysis of Cell Cycle-Related Proteins in CL-6 Cells Treated with 0, 100, 200, and 300 μg/mL of Fucoidan for 48 h. (A) Western blot analysis of cyclin D1 and CDK4 expressions. Relative expression levels of (B) cyclin D1 and (C) CDK4 were evaluated and compared with the control group. * p< 0.05, ** p < 0.01, *** p < 0.001

In Western blot analysis of cell cycle-related protein expression, the results revealed the down-regulation of cyclin D1 and CDK4 in treated cells (Figure 6A). Furthermore, the relative expression level of cyclin D1 and CDK4 were significantly decreased in a concentration-dependent manner (Figure 6B and C).

*Western blot analysis of apoptotic associated proteins expression*

**Figure 7.**
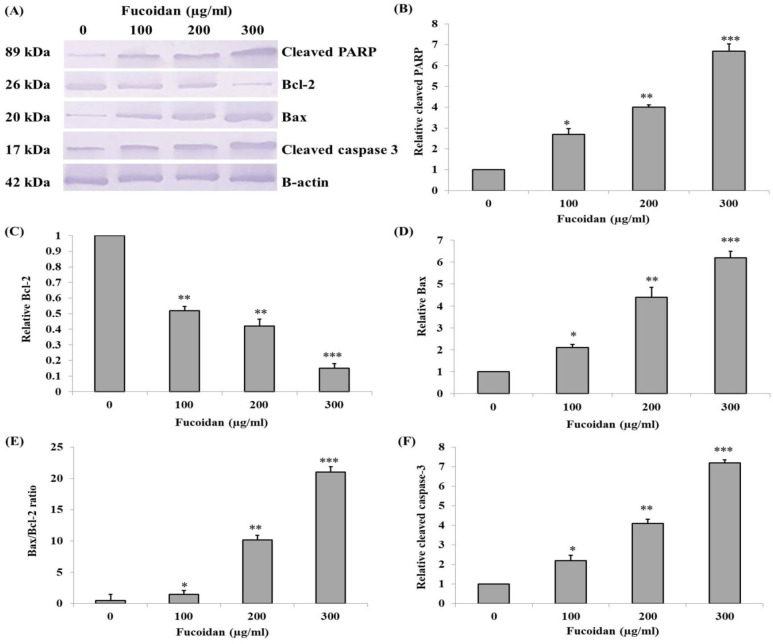
Western Blot Analysis of the Apoptotic Related Protein Expression in CL-6 Cells Treated with 0, 100, 200, and 300 μg/mL of Fucoidan for 48 h. (A) Western blot analysis of cleaved PARP, Bcl-2, Bax, and cleaved caspase-3 expressions. Relative expression levels of (B) cleaved PARP, (C) Bcl-2, (D) Bax, (E) the ratio of Bax and Bcl-2, and (F) cleaved caspase-3 were calculated and compared with the control group. * p< 0.05, ** p < 0.01, *** p < 0.001

Apoptotic effects at the protein expression level of fucoidan in CL-6 cells were also evaluated. After incubation with fucoidan, the expression levels of apoptotic and anti-apoptotic associated proteins were determined. For apoptotic markers, the relative expression level of cleaved PARP was significantly increased in a concentration-dependent manner (Figure 7A and B). The results of apoptotic proteins, Bax (Fig. 7A and D), and cleaved caspase-3 (Figure 7A and F) suggested the significant increase of expression level in a concentration-dependent manner. Oppositely, the expression of the anti-apoptotic protein, Bcl-2, was significantly decreased (Figure 7A and C). Besides, the Bax to Bcl-2 ratio was significantly increased (Figure 7E). 

## Discussion

The unique characteristic of cancer is apoptotic resistance (Hanahan and Weinberg, 2011). Apoptosis can be manifested by the blebbing of the cell membrane with nuclear fragmentation, chromosome condensation, and cytosol shrinking (Burz et al., 2009). Apoptosis is usually categorized into the extrinsic pathway and the intrinsic pathway. The extrinsic pathway associates with the interaction between the death receptor and its ligand meanwhile the intrinsic pathway associates with a disruption in mitochondrial membrane potential by various stimuli. Both pathways finally activate caspase cascades and cause the cell to apoptosis (Brenner and Mak, 2009; Burz et al., 2009; Jeong and Seol, 2008; Khan et al., 2008).

Brown algae contain numerous practically unique sulfated polysaccharides such as alginic acids, laminarans, and fucoidans (Gupta and Abu-Ghannam, 2011). These polysaccharides are used for various purposes especially in the food-processing industry, medicine, and biotechnology (Giavasis, 2014). Several previous studies suggested that fucoidan exerts the growth and proliferation inhibition in human cancer cells via apoptotic induction (Bai et al., 2020; Blaszczak et al., 2018; Chantree et al., 2020; Deepika et al., 2019; He et al., 2019; Liu et al., 2020; Narayani et al., 2019; Palanisamy et al., 2018). Moreover, some studies reported the effect of fucoidan in suppressing the invasion (Lin et al., 2017) and metastasis (Alekseyenko et al., 2007) of cancer cells. Consequently, this present study focused on figure the cytotoxicity effect of fucoidan and demonstrate the related biological molecules on cultured CL-6 cholangiocarcinoma cells.

In the present study, it was revealed that fucoidan induced cytotoxicity in CL-6 cells whereas the normal OUMS cells were not affected. Fucoidan decreased cell viability of CL-6 cells by apoptotic induction as seen in the increasing number of Annexin V positive cells with nuclear chromatin condensation and DNA fragmentation after incubation with fucoidan in a dose-dependent manner. Apoptosis is the one of programmed cell death that involved the cascades of signaling pathways both in the intrinsic and extrinsic pathways. Lastly, there is an activation of the same target apoptotic molecules such as caspase-3 (Jeong and Seol, 2008). The well-known apoptotic marker, Bax, induces apoptosis via mitochondrial membrane potential alteration that results in the translocation of cytochrome c from the mitochondrial inner membrane to the cytosol that triggers signaling of caspase cascades. On the contrary, the anti-apoptotic marker, Bcl-2, prohibits releasing cytochrome c to the cytoplasm that contributes to the suppression of the intrinsic apoptotic pathway (Brenner and Mak, 2009). In Western blot analysis, it demonstrated that fucoidan could induce apoptosis in CL-6 cells as revealed in the increase of Bax but the decrease of Bcl-2 levels, accordingly, increased Bax/Bcl-2 ratio. Previous studies suggested that the increase in the Bax/Bcl-2 ratio suggested the association with mitochondrial dysfunction due to the activation of the intrinsic apoptotic pathway (Hosny et al., 2018).

The active forms of caspases are the critical proteins in apoptotic induction both intrinsic and extrinsic pathways (Stennicke and Salvesen, 1998). In the extrinsic pathway, caspase-8 is stimulated by the binding of the ligands to the death receptors on the cell membrane. Then, caspase-8 cleaves Bid which results in the induction of further caspase cascades. In the intrinsic pathway, it involves the disruption of mitochondrial membrane potential, thus, mitochondrial cytochrome c is released to the cytoplasm that results in caspase-9 activation (Brenner and Mak, 2009). Activated caspase-8 and -9 further activate caspase-7 and caspase-3 which degrade several molecules such as PARP or Poly (ADP-ribose) polymerase. In general, PARP repairs the aberrant DNA. Therefore, PARP is the anti-apoptotic marker, but it serves as a marker of apoptosis when it is degraded (Jin and El-Deiry, 2005). In the present study, the Western blot analysis illustrated that fucoidan up-regulated cleaved form of PARP and cleaved caspase-3 and expression in CL-6 cells in a concentration-dependent manner. 

The alteration of mitochondrial membrane potential (ΔΨm) can cause apoptosis (Philchenkov, 2004). Previous studies revealed that fucoidan induced apoptosis in cancer cells by ΔΨm disruption (Narayani et al., 2019; Park et al., 2011). The present study demonstrated that fucoidan decreased the aggregate to monomer ratio of JC-1 corresponding with the decreased number of cells with intact ΔΨm evaluated by fluorescent microscope. Therefore, according to the effects of fucoidan in the up-regulation of Bax, cleaved caspase-3, and cleaved PARP, down-regulation of Bcl-2, and alteration of ΔΨm, it could be implied that fucoidan has potential in apoptotic induction via an intrinsic pathway on CL-6 cholangiocarcinoma cell. The conclusion can be confirmed by Annexin V/PI flow cytometry analysis that incubation with fucoidan increased the number of apoptotic CL-6 cells in a concentration-dependent manner.

Uncontrolled division is a unique feature of cancer. The mechanism of this characteristic is involved with mutation of cell cycle regulatory genes which allows for a cell to avoid the checkpoint control systems (Hanahan and Weinberg, 2011). It has been revealed that treatment with fucoidan in various types of cell lines could induce cell cycle arrest in different phases. The consolidation of fucoidan with chemotherapeutic agents resulted in cell cycle arrested in the G2/M phase in MCF-7 breast cancer cells (Zhang et al., 2013). Anyhow, in FaDu and H103 head and neck squamous cell carcinoma, treatment with fucoidan leaded cell cycle arrested in S/G2 phase (Blaszczak et al., 2018). In the acute promyelocytic leukemia cell line incubated with fucoidan resulted in cell cycle arrest in the G1 phase (Atashrazm et al., 2016). The same trend of the result was also demonstrated in HT29 colorectal cancer cells, MCF-7 breast cancer cells, and human ovarian cancer cells with downregulation of cyclin D1 and CDK4 (Banafa et al., 2013; Han et al., 2015; Liu et al., 2020). In the present study, we found that treatment with fucoidan in CL-6 cells resulted in cell cycle arrest in the G0/G1 phase. Furthermore, Western blot analysis revealed the decreased expression levels of cyclin D1 and CDK4. The suppression of the PI3K/Akt signaling pathway by fucoidan results in the decreased expression levels of cyclin D1 and CDK4 which induce cell cycle arrest in the G0/G1 phase as described by a recent study (Liu et al., 2020)

In conclusion, the results presented in this study revealed that fucoidan could exert cytotoxicity in CL-6 cholangiocarcinoma via the induction of apoptosis and cell cycle arrest. Nevertheless, future research about the anticancer effect of fucoidan against cholangiocarcinoma should be investigated in other signaling pathways related to its critical characteristics such as migration, invasion, and angiogenesis. Furthermore, the evaluation of fucoidan in combination with chemical agents should be further determined to provide the knowledge of improving the synergistic anticancer potential, with reducing the associated side-effects, against cholangiocarcinoma in the future.

## References

[B1] Alekseyenko T, Zhanayeva SY, Venediktova A (2007). Antitumor and antimetastatic activity of fucoidan, a sulfated polysaccharide isolated from the Okhotsk Sea Fucus evanescens brown alga. Bull Exp Biol Med.

[B2] Alvaro D, Barbaro B, Franchitto A (2006). Estrogens and insulin-like growth factor 1 modulate neoplastic cell growth in human cholangiocarcinoma. Am J Pathol.

[B3] Atashrazm F, Lowenthal RM, Woods GM (2016). Fucoidan suppresses the growth of human acute promyelocytic leukemia cells in vitro and in vivo. J Cell Physiol.

[B4] Back HI, Kim SY, Park SH (2010). Effects of fucoidan supplementation on Helicobacter pylori in humans. FASEB J.

[B5] Bai X, Wang Y, Hu B (2020). Fucoidan induces apoptosis of HT-29 cells via the activation of DR4 and mitochondrial pathway. Mar Drugs.

[B6] Banafa AM, Roshan S, Liu YY (2013). Fucoidan induces G1 phase arrest and apoptosis through caspases-dependent pathway and ROS induction in human breast cancer MCF-7 cells. J Huazhong Univ Sci Technolog Med Sci.

[B7] Bergquist A, Von SE (2015). Epidemiology of cholangiocarcinoma. Best Pract Res Clin Gastroenterol.

[B8] Bertolini M, Sobue T, Thompson A (2017). Chemotherapy induces oral mucositis in mice without additional noxious stimuli. Transl Oncol.

[B9] Bertuccio P, Malvezzi M, Carioli G (2019). Global trends in mortality from intrahepatic and extrahepatic cholangiocarcinoma. J Hepatol.

[B10] Blaszczak W, Lach MS, Barczak W, Suchorska WM (2018). Fucoidan exerts anticancer effects against head and neck squamous cell carcinoma in vitro. Molecules.

[B11] Brenner D, Mak TW (2009). Mitochondrial cell death effectors. Curr Opin Cell Biol.

[B12] Burz C, Berindan-Neagoe I, Balacescu O, Irimie A (2009). Apoptosis in cancer: key molecular signaling pathways and therapy targets. Acta Oncol.

[B13] Chantree P, Surarak T, Sangpairoj K (2020). Antitumor effects of fucoidan via apoptotic and autophagic induction on HSC-3 oral squamous cell carcinoma. Asian Pac J Cancer Prev.

[B14] Chen T, Wong YS, Zheng W, Bai Y, Huang L (2008). Selenium nanoparticles fabricated in Undaria pinnatifida polysaccharide solutions induce mitochondria-mediated apoptosis in A375 human melanoma cells. Colloids Surf B Biointerfaces.

[B15] Coombe DR, Parish CR, Ramshaw IA, Snowden JM (1987). Analysis of the inhibition of tumor metastasis by sulphated polysaccharides. Int J Cancer.

[B16] Deepika MS, Thangam R, Sheena TS (2019). A novel rutin-fucoidan complex based phytotherapy for cervical cancer through achieving enhanced bioavailability and cancer cell apoptosis. Biomed Pharmacother.

[B17] Dinesh S, Menon T, Hanna LE (2016). In vitro anti-HIV-1 activity of fucoidan from Sargassum swartzii. Int J Biol Macromol.

[B18] Fouassier L, Marzioni M, Afonso MB (2019). Signalling networks in cholangiocarcinoma: Molecular pathogenesis, targeted therapies and drug resistance. Liver Int.

[B19] Giavasis I (2014). Bioactive fungal polysaccharides as potential functional ingredients in food and nutraceuticals. Curr Opin Biotechnol.

[B20] Geert VW, Marcin B, Karolina O (2019). Fucoidan structure and activity in relation to anti-cancer mechanisms. Mar Drugs.

[B21] Gupta S, Abu-Ghannam N (2011). Bioactive potential and possible health effects of edible brown seaweeds. Trends Food Sci Technol.

[B22] Han YS, Lee JH, Lee SH (2015). Antitumor effects of fucoidan on human colon cancer cells via activation of Akt signaling. Biomol Ther.

[B23] Hanahan D, Weinberg RA (2011). Hallmarks of cancer: the next generation. Cell.

[B24] He X, Xue M, Jiang S (2019). Fucoidan promotes apoptosis and inhibits EMT of breast cancer cells. Biol Pharm Bull.

[B25] Hosny G, Ismail W, Makboul R, Badary FAM, Sotouhy TMM (2018). Increased glomerular Bax/Bcl2 ratio is positively correlated with glomerular sclerosis in lupus nephritis. Pathophysiology.

[B26] Hou Y, Wang J, Jin W, Zhang H, Zhang Q (2012). Degradation of Laminaria japonica fucoidan by hydrogen peroxide and antioxidant activities of the degradation products of different molecular weights. Carbohydr Polym.

[B27] Huang TH, Chiu YH, Chan YL (2015). Prophylactic administration of fucoidan represses cancer metastasis by inhibiting vascular endothelial growth factor (VEGF) and matrix metalloproteinases (MMPs) in Lewis tumor-bearing mice. Mar Drugs.

[B28] Imbs T, Skriptsova A, Zvyagintseva T (2014). Antioxidant activity of fucose-containing Sulfated polysaccharides obtained from Fucus Evanescens by different extraction methods. J Appl Phycol.

[B29] Jeong SY, Seol DW (2008). The role of mitochondria in apoptosis. BMB Rep.

[B30] Jeong YT, Kim YD, Jung YM (2013). Low molecular weight fucoidan improves endoplasmic reticulum stress-reduced insulin sensitivity through AMP-activated protein kinase activation in L6 myotubes and restores lipid homeostasis in a mouse model of type 2 diabetes. Mol Pharmacol.

[B31] Jin Z, El-Deiry WS (2005). Overview of cell death signaling pathways. Cancer Biol Ther.

[B32] Khan N, Adhami VM, Mukhtar H (2008). Apoptosis by dietary agents for prevention and treatment of cancer. Biochem Pharmacol.

[B33] Khan SA, Davidson BR, Goldin RD (2012). Guidelines for the diagnosis and treatment of cholangiocarcinoma: an update. Gut.

[B34] Kim EJ, Park SY, Lee JY, Park JH (2010). Fucoidan present in brown algae induces apoptosis of human colon cancer cells. BMC Gastroenterol.

[B35] Lee HR, Yoo N, Kim JH (2016). The therapeutic effect of PLAG against oral mucositis in hamster and mouse model. Front Oncol.

[B36] Leelawat K, Leelawat S, Tepaksorn P (2006). Involvement of c-Met/hepatocyte growth factor pathway in cholangiocarcinoma cell invasion and its therapeutic inhibition with small interfering RNA specific for c-Met. J Surg Res.

[B37] Li B, Lu F, Wei X, Zhao R (2008). Fucoidan: Structure and Bioactivity. Molecules.

[B38] Lin J, Wang K, Wang H (2017). Fucoidan reduced the invasion of oral squamous cell carcinoma cells and modified their effects to macrophages. Med Oncol.

[B39] Liu S, Yang J, Peng X, Li J, Zhu C (2020). The natural product fucoidan inhibits proliferation induces apoptosis of human ovarian cancer cells: focus on the PI3K/Akt signaling pathway. Cancer Manag Res.

[B40] Mabeau S, Kloareg B, Joseleau JP (1990). Fractionation and analysis of fucans from brown algae. Phytochemistry.

[B41] Mancino A, Mancino MG, Glaser SS (2009). Estrogens stimulate the proliferation of human cholangiocarcinoma by inducing the expression and secretion of vascular endothelial growth factor. Dig Liver Dis.

[B42] Mansour MB, Balti R, Yacoubi L (2019). Primary structure and anticoagulant activity of fucoidan from the sea cucumber Holothuria polii. Int J Biol Macromol.

[B43] Narayani SS, Saravanan S, Ravindran J, Ramasamy M, Chitra J (2019). In vitro anticancer activity of fucoidan extracted from Sargassum cinereum against Caco-2 cells. Int J Biol Macromol.

[B44] Ogasawara S, Yano H, Higaki K (2001). Expression of angiogenic factors, basic fibroblast growth factor and vascular endothelial growth factor, in human biliary tract carcinoma cell lines. Hepatol Res.

[B45] Palanisamy S, Vinosha M, Manikandakrishnan M (2018). Investigation of antioxidant and anticancer potential of fucoidan from Sargassum polycystum. Int J Biol Macromol.

[B46] Park HS, Kim GY, Nam TJ, Deuk Kim N, Hyun Choi Y (2011). Antiproliferative activity of fucoidan was associated with the induction of apoptosis and autophagy in AGS human gastric cancer cells. J Food Sci.

[B47] Park JO, Oh DY, Hsu C (2015). Gemcitabine plus cisplatin for advanced biliary tract cancer: A systematic review. Cancer Res Treat.

[B48] Philchenkov A (2004). Caspases: potential targets for regulating cell death. J Cell Mol Med.

[B49] Phull AR, Kim SJ (2017). Fucoidan as bio-functional molecule: Insights into the anti-inflammatory potential and associated molecular mechanisms. J Funct Foods.

[B50] Ramírez-Merino N, Aix SP, Cortés-Funes H (2013). Chemotherapy for cholangiocarcinoma: An update. World J Gastrointest Oncol.

[B51] Rodríguez-Jasso RM, Mussatto SI, Pastrana L, Aguilar CN, Teixeira JA (2013). Extraction of sulfated polysaccharides by autohydrolysis of brown seaweed Fucus vesiculosus. J Appl Phycol.

[B52] Rui X, Pan HF, Shao SL, Xu XM (2017). Anti-tumor and anti-angiogenic effects of fucoidan on prostate cancer: possible JAK-STAT3 pathway. BMC Complement Altern Med.

[B53] Sapisochin G, Facciuto M, Rubbia-Brandt L (2016). Liver transplantation for “very early” intrahepatic cholangiocarcinoma: International retrospective study supporting a prospective assessment. Hepatology.

[B54] Sapisochin G, Rodríguez DLC, Gastaca M (2014). ”Very early” intrahepatic cholangiocarcinoma in cirrhotic patients: should liver transplantation be reconsidered in these patients?. Am J Transplant.

[B55] Socoteanu MP, Mott F, Alpini G, Frankel AE (2008). c-Met targeted therapy of cholangiocarcinoma. World J Gastroenterol.

[B56] Stennicke HR, Salvesen GS (1998). Properties of the caspases. Biochim Biophys Acta.

[B57] Valle J, Wasan H, Palmer DH (2010). Cisplatin plus gemcitabine versus gemcitabine for biliary tract cancer. N Engl J Med.

[B58] Valle JW, Borbath I, Khan SA (2016). Biliary cancer: ESMO clinical practice guidelines for diagnosis, treatment and follow-up. Ann Oncol.

[B59] Xue M, Ge Y, Zhang J (2012). Anticancer properties and mechanisms of fucoidan on mouse breast cancer in vitro and in vivo. PLoS One.

[B60] Zhang Z, Teruya K, Yoshida T, Eto H, Shirahata S (2013). Fucoidan extract enhances the anti- cancer activity of chemotherapeutic agents in MDA-MB-231 and MCF-7 breast cancer cells. Mar Drugs.

[B61] Zhuang C, Itoh H, Mizuno T, Ito H (1995). Antitumor active fucoidan from the brown Seaweed, Umitoranoo (Sargassum thunbergii). Biosci Biotechnol Biochem.

